# Preparing care home staff to manage challenging behaviours among residents living with dementia: A mixed-methods evaluation

**DOI:** 10.1177/2055102920933065

**Published:** 2020-07-03

**Authors:** Niyah Campbell, Ian D Maidment, Emma Randle, Rachel L Shaw

**Affiliations:** 1 University of Birmingham, UK; 2 Aston University, UK; 3 Unite the Union, UK

**Keywords:** dementia, medication therapy management, nursing homes, patient-centred care, psychological intervention

## Abstract

We evaluated an intervention designed to manage challenging behaviours of people with dementia. Framework analysis of interviews (*n* = 21) showed the intervention modified practice and perceptions. The intervention (*n* = 58; power calculation proposed *n* = 160 for medium effect) had no significant effect on attitudes to dementia for time (*p* = .42) or care home (*p* = .15). The Maslach burnout scores did not change significantly for person-centredness for time (*p* = .83) or care home (*p* = .29). Hope scores showed a significant effect post-intervention (*p* = .004), but this was not maintained. No significant main effect was found for care home (*p* = .36). Experiential learning enabled staff to experience benefits of person-centred care firsthand.

## Background and objectives

There are approximately 280,000 people living with dementia residing in care homes in the United Kingdom (Alzheimer’s Society, 2016). With the prevalence of dementia continually increasing, steps must be taken to ensure that the right care and support is available, including conducting research with care homes. Care home research is a priority for the UK’s National Institute for Health Research (NIHR Dissemination Centre, 2017).

A key issue faced in the care of people with dementia living in care homes is the management of behaviours that challenge, otherwise referred to as Behavioural and Psychological Symptoms of Dementia. Behaviours that challenge associated with dementia include agitation, wandering, shouting, biting and aggression and are broadly considered to be any behaviour that is dangerous to the person or others (Andrews, 2006).

Behaviours that challenge are often managed using psychotropic drugs such as antipsychotics, antidepressants, mood stabilisers and benzodiazepines and other sedatives. The UK *National Dementia Strategy* estimated that 180,000 people with dementia were being prescribed antipsychotic medication (Banerjee, 2009). The review argued that antipsychotic medications were over-prescribed as only 36,000 people were thought to benefit from them. Furthermore, an additional 1800 deaths and 1620 cerebrovascular adverse events per year were directly attributed to their use (Banerjee, 2009). Subsequent reports by the Royal Pharmaceutical Society have supported this notion by highlighting risks associated with the prescription of psychotropics to people with dementia and the benefits of training care staff in non-pharmacological approaches to managing behaviours that challenge (Dementia Action Alliance and Royal Pharmaceutical Society, 2009; Wales Royal Pharmaceutical Society, 2016).

MEDREV was a combined pharmacy/health psychology intervention developed for a feasibility study which aimed to improve the care of People Living With Dementia (PLWD) living in care homes (Maidment et al., 2016, 2018, 2020). The pharmacy component of the study was a medication review, informed by earlier work (Child et al., 2012), designed to target and reduce, where appropriate, the prescription of psychotropics for the management of behaviours that challenge. The health psychology component was a staff training intervention designed to prepare care home staff to manage behaviours that challenge using non-pharmacological methods.

In this article, we report a mixed-methods evaluation of the health psychology intervention directed at care staff. (The feasibility data and main findings of the MEDREV study have been previously reported (Maidment et al., 2018, 2020).) In brief, the intervention was designed to improve the management of behaviours that challenge by training care staff: (1) to respond to behaviours that challenge as an expression of unmet need and (2) to communicate with compassion; we aimed to achieve this by providing care staff with additional skills for person-centred care.

The research question for the qualitative component of the evaluation was: what are care home staffs’ experiences of the training intervention developed to prepare them for the management of behaviours that challenge among residents with dementia. The hypotheses tested in the quantitative component were as follows: hyp 1 – the training intervention will improve attitudes towards dementia and hyp 2 – the training intervention will decrease staff burnout.

## Methods

A mixed-methods design was used to evaluate the intervention. Ethical approval was received as part of the larger MEDREV study (reference no. 15/EM/0314). First, the setting in which the intervention took place will be described. Second, the details of the training intervention workshops will be provided to enable the reader to understand more fully the nature of the intervention being evaluated.

### Participants and setting

Care homes in the local trust with at least 40 residents were invited to take part. Care home staff were paid their hourly rate to attend the training intervention (attendance was limited to 16, but numbers varied across homes). Managers of care homes and care staff (nursing staff, professional carers and activity coordinators) and General Practitioners (GPs) directly involved in the medication review (Maidment et al., 2016) were invited to participate.

### Training intervention with care staff

The care staff intervention involved a 3-hour educational workshop entitled *Inside Out*; repeated twice at each care home. Sessions were facilitated by (NC and RLS).

The workshop was designed to prioritise experiential learning and be interactive, drawing on the expertise and experience of care staff in attendance. The sessions involved different activities including a PowerPoint presentation, a workbook (available on request from corresponding author), group discussions, videos and role plays.

Sessions began with a brief overview of the study, key evidence of the over-prescription of psychotropics and best practice guidance for the use of non-pharmacological approaches to manage behaviours that challenge (Banerjee, 2009; Wales Royal Pharmaceutical Society, 2016). The nature of behaviours that challenge, soliciting examples from care staff’s experience, was then discussed.

Next, person-centred care was introduced (Kitwood, 1988, 1997) as an approach for managing, reducing and potentially preventing behaviours that challenge. This was consolidated into more tangible techniques with Brooker’s *VIPS* framework: Valuing Personhood, Individualised Needs, Personal Perspectives and Social Environment (Brooker, 2004; Houghton et al., 2016; Røsvik et al., 2011). Video materials played mock interactions between care staff and people with dementia. One example portrayed two approaches to a scenario in which a carer attempted to dress a resident (Passalacqua and Harwood, 2012): (1) a task-focused approach in which the carer communicated with the resident in a hurried fashion and used ‘elderspeak’ (Williams et al., 2003) – that patronising tone which ignores personhood and obscures individual needs and (2) a person-centred approach in which the carer patiently communicated with the resident and provided opportunity for them to express their personal wishes.

**Figure 1.**
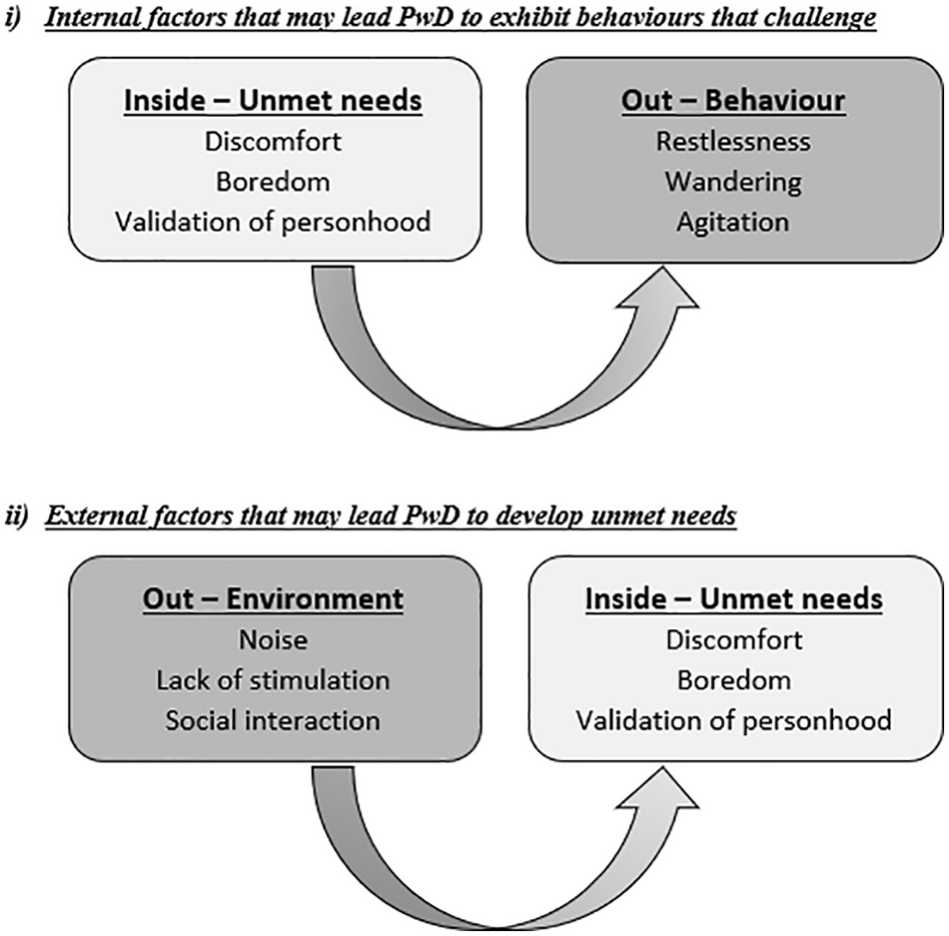
An illustrative example of ‘Inside Out Thinking’.

Following this, the notion of ‘Inside Out Thinking’ was introduced (see Figure 1). Behaviours that challenge exhibited by people with dementia often reflect what is going on *internally* but cannot be easily expressed; *external* factors play a key role in the presentation and resolution of behaviours that challenge because they affect how people with dementia feel which, in turn, effects how they act. Inside Out Thinking was developed by (author initials) to stress the importance of the target behaviour – recognising behaviours that challenge is an expression of unmet (physical, emotional and/or psychosocial) need. Inside Out Thinking encourages the use of an investigative approach when faced with behaviours that challenge in order to identify potential triggers, determine mechanisms for removing triggers and provide beneficial distractions for people with dementia which meet their individual needs and preferences.

To conclude, key features of an effective, person-centred team were discussed. Clear and open channels of communication were emphasised, both in relation to people with dementia and also positive and supportive interactions between staff (Gilster et al., 2018). The importance of self-care was stressed; providing person-centred care to people with dementia is demanding and care staff need to look after their own well-being in order to effectively manage that of their residents. All attendees were given a pen with the VIPS embossed along the side as a small environmental cue. Additional pens and workbooks were provided to care homes for staff who had been unable to attend.

Following the workshop, (author initials) conducted 2 monthly follow-up visits to each care home. During these visits, discussions were held with care home managers and care staff to identify ways of maintaining awareness of the workshop content among those who had attended and spread to those who had not. Recommendations we made as a component of the intervention included the following: displaying content-related posters and slogans on staff noticeboards; the inclusion of person-centred–related questions in staff supervisions/appraisals and meetings; and increasing efforts to learn about residents’ social histories through engagement with family members and ‘About Me’ books.

### GP training

GPs involved were provided with a summary of the care staff training intervention and were invited to contact the research team should they wish further information. To augment the written material, a short (20 minutes) training session about evidence-based recommendations to reduce the use of psychotropic medication for the management of behaviours that challenge was delivered to GPs by (authors initials) or the pharmacist that delivered the medication review, either face-to-face or by phone.

### Data collection

#### Semi-structured interviews

Individual semi-structured interviews were conducted with care home managers, care staff and GPs at two timepoints: in the week preceding the intervention and 3 months post-intervention. The interview schedule (see supplementary file 1) aimed to gather data on participants’ perceptions of behaviours that challenge, approaches taken to manage behaviours that challenge and feedback on the intervention. Questions included, for example: Care staff: without telling me who the individual is, can you describe a time when you tried out a person-centred approach and it worked really well? Have you now tried this with others/in other situations?; Manager: what do you anticipate the barriers to implementing this intervention will be in your care home?; and GP: tell me about your approach to people with dementia generally in your practice? All interviews were conducted by (author initials). Written consent was obtained prior to all interviews, which were audio-recorded and transcribed verbatim.

#### Measures

Care staff who received the training intervention completed: the Approaches to Dementia Questionnaire (ADQ) (Lintern et al., 2000) and the Maslach Burnout Inventory (MBI) Health Services Survey (Maslach and Jackson, 1981). Questionnaires were administered at three timepoints: (T1) pre-intervention baseline: ADQ and MBI; (T2) immediately post-intervention: ADQ; and (T3) 3 months post-intervention: ADQ and MBI. All participants were briefed by (insert initials) and provided with a Participant Information Sheet before written consent was obtained. A power calculation indicated a sample of *n* = 160 would be required to detect medium-sized effects.

Questionnaires at T1 and T2 were administered by (author initials). At T3, (author initials) provided care home administrative staff with named, sealed envelopes containing instructions and questionnaire materials. Administrative staff distributed envelopes to participants, who were asked to complete measures, seal them inside a blank envelope and return to administrative staff for later collection by the researcher.

### Data analysis

#### Framework analysis of interview data

Interview data were analysed using the framework analysis (Ritchie and Spencer, 1994). Themes were developed from both the research questions (deductive) and the interview narratives (inductive) (Pope et al., 2000). Data were charted into a data matrix using the Microsoft^®^ Excel (Swallow et al., 2003).

#### Analysis of measures

Data gathered from both measures at all timepoints were entered into the statistical program SPSS 21.

#### Approaches to Dementia Questionnaire

Ten of the 19 items were reverse coded as per instructions. A total score (possible score 19–95), and subscales for ‘hope’ (possible score 8–40) and ‘person-centredness’ (possible score 11–55) were calculated. To test the hypothesis that training improved attitudes towards dementia, a repeated measures analysis of variance (ANOVA) was conducted to identify significant changes in ADQ total score, and person-centredness and hope subscales at T2 and T3 compared to T1.

#### The Maslach Burnout Inventory: Human Services Survey

Three subscales were calculated from the 22-item scale to create a score for emotional exhaustion, depersonalisation and personal accomplishment. To test the hypothesis that the training decreased burnout, a series of paired *t*-tests was conducted to determine whether individuals receiving the training reported reduced burnout scores at T3 compared to T1.

## Results

Five care homes were recruited (see Table 1).

**Table 1.** Characteristics of care homes.

Care home ID	Type of service, medication management and training-related information
CH1	• Care home with nursing. • Medication managed by qualified nurses. • Care home staff provided with ‘Dignity in Care Training’.
CH2	• Residential care only. • Medication managed by care team with medication training. • Management and senior staff had undergone expert VIPS training at Worcester University.
CH3	• Care home with nursing. • Medication managed by qualified nurses. • Care home staff had undergone Dementia Tour Training.
CH4	• Care home with nursing. • Medication managed by qualified nurses. • All care home staff were undergoing an extensive Person-Centred Care-related training scheme at time of enrolment onto MEDREV.
CH5	• Care home with nursing. • Medication managed by qualified nurses. • Custom, behavioural training provided to care home staff by care home manager.

### Framework analysis of interview data

Interviews were conducted with five care home managers, 13 care staff and three GPs (see Table 2).

**Table 2.** Interviewee characteristics.

Participant ID	Gender	Affiliated care home	Interviewed pre-intervention	Interviewed post-intervention	Job title
Care staff					
CS1	M	CH1	Y	Y	Carer
CS2	F	CH1	Y	Y	Senior carer
CS3	F	CH1	Y	Y	Carer
CS4	F	CH1	Y	Y	Carer
CS5	F	CH2	Y	Y	Senior carer
CS6	F	CH2	Y	Y	Senior carer
CS7	F	CH2	Y	Y	Carer
CS8	F	CH3	Y	N	Carer
CS9	F	CH3	Y	N	Senior carer
CS10	F	CH3	Y	Y	Carer
CS11	F	CH4	Y	Y	Carer
CS12	F	CH4	Y	Y	Nurse
CS13	F	CH5	Y	Y	Nurse
Care home managers					
CHM1	F	CH1	Y	Y	–
CHM2	F	CH2	Y	Y	–
CHM3	F	CH3	Y	Y	–
CHM4	M	CH4	Y	Y	–
CHM5	M	CH5	Y	Y	–
General Practitioners					
GP1	M	CH1	Y	Y	–
GP2	F	CH3	N	Y	–
GP3	M	CH2	N	Y	–

Findings are presented under the themes identified: defining behaviours that challenge, providing person-centred care, medication use and intervention-related feedback.

#### Defining behaviours that challenge

Care staff most commonly associated the term ‘behaviours that challenge’ with aggressive behaviours directed towards themselves or others (e.g. physically striking out or use of abusive language). Aggressive behaviour was most frequently encountered when providing personal care to residents with dementia (e.g. administering medication and assisting with bathing). Non-aggressive behaviours – such as wandering and persistent noise-making – also challenged staff as these behaviours were often disruptive to other residents and/or the day-to-day caregiving routine. It was feelings of frustration, fear and uncertainty (of how to respond) that underpinned perceptions of these behaviours as ‘challenging’:

. . . you say ‘come on, can you come?’ ‘I don’t want to go!’ or something and you, it’s trying to get round that and some have sort of hit out or got angry, I find that a bit challenging . . . because . . . I don’t always know really the best way to [manage it]. (Care staff (CS)3, pre-intervention)

Most care staff demonstrated an understanding of the role that unmet needs played in leading to behaviours that challenge. It was felt that such behaviours were often an expression of unmet needs that people with dementia had difficulty communicating due to a *‘communication deficit’* (CS13) caused by dementia:

. . . they used to get up in the morning, go to work, come home, look after their children, do their duties. Now, when they [have] dementia they can’t do all those things and they get angry, they get aggressive . . . they try to tell you that they can’t do things that they used to but it comes out as aggression and challenging. (CS9, pre-intervention)

Within workshop sessions, most of the views expressed by care staff on what behaviours that challenge were and why residents with dementia exhibited them aligned with the messages promoted by the ‘Inside Out’ intervention. For this reason, post-intervention, many participants’ views were unchanged. Nevertheless, participants felt to have benefitted from participation as the session had heightened awareness of what people with dementia are capable of, why they present behaviours that challenge and how care practice affects them:

It’s always good to get a refresher . . . because it makes our clients people again . . . I think that’s the worst thing, is when you start looking at clients as work rather than people and the training like puts it into perspective. (CS13, post-intervention)

For those who had previously undervalued the role that unmet needs play in the presentation of behaviours that challenge, participation in ‘Inside Out’ had been an informative experience. Discussions held within the workshop explored potential causes of behaviours that challenge which had, in turn, changed perceptions:

Before . . . anything would be challenging behaviour to me . . . from them being very vocal in the lounge . . . verbally aggressive and physically aggressive . . . whereas now, I have to look into as in ‘it’s not really challenging behaviour’ . . . there’s a reason behind . . . their behaviour. (CS10, post-intervention)

This example displays a shift in perception from behaviours that challenge being problematic, trivial and attributable to the individual, to something approaching meaningful behaviours that hold a message in need of decoding.

#### Providing person-centred care

Responses to questions about daily work practices indicated that a compassionate, person-centred approach was broadly taken by care staff when caring for residents with dementia. Care staff valued residents’ personhood, acknowledged their individual identities and provided *‘tailored care’* (CS2). This displayed an understanding of personal perspectives through empathic actions such as creating a positive social environment by personalising living spaces and providing meaningful activities:

They had a life before . . . they was doctors . . . teachers . . . lawyers . . . you have to learn to respect them and remember that they’ve not just came like this. (CS1, pre-intervention)

I go with the flow and talk because to them they’re – whatever they’re talking about, that’s what’s happening to them at the time . . . because I can’t imagine what it’s like for someone to be told something’s not happening that they truly believe is, it must be vile. (CS5, pre-intervention)

When asked how they typically managed behaviours that challenge, care staff reported use of a wide range of behavioural approaches. The primary approach to address behaviours that challenge was verbal communication. A *‘soft’* (CS2), *‘calm’* (CS4) tone was used when verbally engaging residents were agitated or upset. They understood that this made them appear non-oppositional and supportive which soothed residents. Other techniques included the use of distraction techniques, altering the environment or simply providing residents with space and monitoring them from a distance:

It may be that they want something . . . offer them like, a drink or food or ask them to come and sit in a quieter place. You just try to calm them down that way because they can’t explain what they want sometimes so you have to try and work different things . . . (CS7, pre-intervention)

Care staff acknowledged the need to be pro-active. Processes such as charting antecedents and consequences of challenging behaviour and holding focused discussions in staff meetings/handovers allowed them to better share information about residents and their behaviours. Care staff used this information to identify and reduce exposure to triggers of behaviours that challenge:

. . . nine times out of ten there is a trigger for it so it’s more trying to remove any triggers before it actually happens . . . (CS6, pre-intervention)

Although care home managers felt that most staff engaged in person-centred care, they did report that shortfalls in its provision were not uncommon, for example:

. . . they talk about their parents, she miss her granny . . . If you explain them that – ‘you are ninety-three years old, you’ve got no parents’, try to like go backwards and just say ‘listen, you are ninety-three years old, you think about how old your parents are’. (CS8, pre-intervention)

Such shortfalls were of significant concern to managers as, on occasion, non-person-centred practice was identified as the antecedent to ‘challenging behaviour’. In some instances, lack of person-centredness was attributed to staff not having undergone relevant training; this was felt to be the case particularly for those new to residential care and staff whose roles were focused on medication management rather than direct care:

The lack of behavioural training in the nursing is a worry . . . A lot of these nurses haven’t done any basic form of behavioural training . . . (Care home manager (CHM)5, pre-intervention)

Limitations to time and resources were regarded to be a second significant barrier to person-centredness; time required to complete physical tasks was often perceived to compete directly with that spent interacting with residents. Such beliefs led care staff to adopt task-orientated attitudes, which were only exacerbated by the high workloads and low staffing levels typical of the residential care sector. Despite disapproving of task-orientated attitudes, managers empathised with the pressures on staff:

The majority of the time you’d try to persuade, find out what it is they’re trying to do to try and understand but I think . . . sometimes . . . they just don’t have the time . . . (CHM3, post-intervention)

Emotional burnout was associated with delivering person-centred care because it was perceived as being emotionally labour-intensive and sometimes ineffective. Managers felt that repeated failures to successfully manage behaviours that challenge this way occasionally led care staff to feel *‘frustrated’* (CHM3) and incompetent. When this occurred, medical solutions were often sought:

. . . I was reading the [MEDREV] study, I remember thinking ‘absolutely essential piece of work’ because it’s not care professionals being lazy, it’s usually care professionals not knowing the answers and looking for the answer in a bottle. (CHM5, pre-intervention)

When asked if the intervention had made a difference, responses among care staff were mixed; some saw no significant difference, but others had observed notable changes. Although few staff could remember what the VIPS acronym stood for, there was clear understanding of the key messages and techniques provided. The workshop was described as a *‘refresher’* (CS13) of the importance of providing person-centred care which had given staff a renewed sense of agency:

And just driving it through with them, that everybody’s an individual, treat them (as such). (CHM3, post-intervention)

For some, renewed vigour for person-centred care was credited to the way the intervention had illustrated its merits for both residents and staff alike. This example displays elements of self-care and support within the team, as well as the realisation of the benefits of providing person-centred care:

They’ve got to get through so much work . . . that would impact on how they perceive what they can do with the clients but what was great was that it really showed up that it takes less time . . . to allow the client to have choice, for them to feel more validated and for them to feel good about their day. (CS13, post-intervention)

All managers reported to have observed positive changes in care practice and believed that the VIPS framework effectively conveyed the benefits of person-centred care:

I really do think they’ve embraced it. It’s took a long time, a lot of hard work but I think . . . they understand now the importance of not approaching someone from behind, not standing, whispering in the corner and causing someone to be paranoid . . . it’s definitely a lot better. (CHM3, post-intervention)

This theme detected the presence of person-centredness, and also identified barriers to its provision. Changes in care staff behaviour were evidenced following the intervention, demonstrating its success. Furthermore, the consolidation of the abstract notion of person-centred care into the VIPS and ‘Inside Out Thinking’ facilitated this change.

#### Medication use

Medication was managed by registered nurses in four of the five participating care homes. Consequently, knowledge of psychotropic medication among care staff was often vague as they had little-to-no involvement in medication-related processes. Most care staff simply understood psychotropics as a form of medication given to people with dementia to *‘calm them down’* (CS2). Negative side effects such as drowsiness and falls had been observed, but psychotropics were viewed as an *‘easy’* solution:

They’re a mental health drug . . . I understood that people living with dementia wasn’t supposed to be treated by half of them. But, it’s an easy way of trying to handle sometimes, where doctors are concerned. And in my own experience, sometimes they’re too easy to get hold of, and increase. So, but I do know that they’re supposed to help calm, relax and – but then they can also increase the level of falls and accidents that happen. (CS6, pre-intervention)

Care home managers were unanimous in believing that medications used to manage behaviours that challenge were the *‘last resort after you’ve tried all other avenues’* (CHM2):

The last thing that we do is the ‘chemical cosh’, is give antipsychotics because even people that walk around the home, if you sedate them, that you increase the risk of falls ten-fold. (CHM1, pre-intervention)

In some homes, prior attempts to reduce medication had failed due to a perceived reluctance among medical professionals to make changes to long-standing prescriptions:

I was planning to review five patients in one surgery then I had a chance to spoke to the GP with the three patients, the GP said ‘they are being with the tablet for a long time . . . there is no need for any changes so just continue as it is’. (CS12, pre-intervention)

Some care staff felt anxious prior to the medication review as they believed that changes to medications would result in increased prevalence of behaviours that challenge. However, the educational workshop effectively addressed these concerns by outlining the supporting evidence behind MEDREV:

At first . . . a lot of us were like ‘Really? What’s it gonna cause?’ but . . . having that bit of training alone about . . . person-centred care . . . it has changed people’s views. (CS11, post-intervention)

Your training . . . alleviated some of the fears . . . people were anticipating you take them off the medication and you’re still gonna see the aggression, it’s gonna come back twice as bad . . . whereas your training, workshop was to open your mind to say ‘well, how do you know that?’. (CHM4, post-intervention)

Post-intervention, some managers witnessed a reduced eagerness of staff to seek medication to manage behaviours that challenge, because the intervention boosted care staff’s self-efficacy to manage behaviours that challenge through non-pharmacological methods:

The most constructive bit for me, is the thinking more about the individual and what we can do as opposed to what I can reach for to give. And that’s probably been one of the bigger changes. (CHM5, post-intervention)

One of the GPs confirmed this view that medication could be over-used in the treatment of behaviour that challenges:

Because sometimes they’re overused in the treatment of dementia. (GP3, post-intervention)

Following the intervention, there was a perceived reduction in primary care workload, because the care staff were more confident in managing behaviour that challenges with less reliance on medication:

There was a reduced number of calls . . . (before we were) . . . getting lots of calls that seemed fairly minor, where we going and weren’t necessarily doing anything very active and it was more reassurance rather than anything else . . . but subjective impression was that the number went down . . . everybody that has been involved has thought of it very positive with trying to decrease medication. (GP2, post-intervention)

#### Intervention-related feedback

Managers reported having experienced no practical issues in accommodating the ‘Inside Out’ workshop. Feedback from attendees was largely positive. Care staff described the workshop as *‘interesting’, ‘informative’* and *‘useful’* (CS8, 9, 11 and 13) with a *‘good balance of participation and teaching’* (CS13). These views were echoed by managers, who said that the workshops *‘were all positive’* (CHM4) and *‘really helpful’* (CHM2).

A highly valued attribute of the intervention was the trainer’s own professional experience of residential care. This meant that real-life examples that resonated with staff could be shared; equally, it meant that the trainer could empathise more readily with challenges faced by staff. This made for a relaxed trainer–trainee dynamic and created a safe space in which care-related matters could be discussed openly:

I think it was geared at them and because you’re from that background . . . they understood it . . . Sometimes you’ll get trainers that’ll think that there’s an answer to everything in every situation and sometimes there isn’t an answer . . . if you’ve actually done the job then you know it doesn’t always happen like that . . . you can be more honest and say . . . ‘I’ve never come across that before but let’s talk about it’. (CHM1, post-intervention)

### Statistical analysis of measures

In total, 142 care staff participated in the ‘Inside Out’ workshop across five care homes. Complete questionnaire data sets were received from 58 (41% of the sample). This meant that our analyses were underpowered, meaning findings must be interpreted with caution. Nevertheless, the findings helped describe the characteristics of the sample.

A repeated measures ANOVA was conducted to assess whether the intervention improved care staff attitudes towards dementia at T2 and T3 compared to T1. Time was the within-subjects condition, and care home was the between-subjects condition. Table 3 displays the mean total ADQ scores and standard deviations (SDs) for each time point. No significant main effects were found for either time (*F*(2, 106) = .879, *p* = .42, $\eta_{p}^{2}=.02$ ) or care home (*F*(4, 53) = 1.79, *p* = .15, $\eta_{p}^{2}=.12$ ). The interaction between time and care home was also found to be non-significant (*F*(8, 106) = 1.12, *p* = .36, $\eta_{p}^{2}=.08$ ), suggesting that receiving the training did not result in significantly improved attitudes to dementia within any of the care homes at any time point.

**Table 3.** Total, person-centredness and hope scores for the ADQ measured prior to training (baseline), immediately post-training and 3 months post-training.

		ADQ total score M (SD)			ADQ person-centred M (SD)			ADQ hope score M (SD)		
	*N*	Baseline	Immediately post-training	3 months post-training	Baseline	Immediately post-training	3 months post-training	Baseline	Immediately post-training	3 months post-training
Care home										
CH1	8	79.75 (5.20)	76.13 (16.30)	76.25 (13.49)	51.50 (2.27)	45.63 (16.30)	49.25 (5.01)	28.25 (3.69)	30.50 (4.38)	27.00 (9.07)
CH2	4	80.75 (6.60)	83.00 (9.06)	84.75 (3.59)	50.00 (4.24)	51.00 (5.23)	54.00 (1.41)	30.75 (6.80)	32.00 (5.72)	30.75 (3.40)
CH3	15	66.87 (14.67)	75.00 (8.92)	71.53 (15.58)	44.00 (11.45)	47.33 (8.53)	45.20 (13.60)	22.87 (6.08)	27.67 (5.70)	26.33 (4.42)
CH4	19	76.26 (8.07)	76.16 (14.24)	74.53 (11.54)	50.00 (3.09)	47.63 (9.52)	48.11 (4.65)	26.26 (7.26)	28.53 (7.41)	26.42 (7.93)
CH5	12	76.58 (4.87)	80.83 (6.46)	71.67 (14.50)	50.83 (3.27)	50.92 (3.20)	46.00 (12.63)	25.75 (3.17)	29.92 (5.92)	25.67 (4.33)

M: mean; SD: standard deviation; ADQ: Approaches to Dementia Questionnaire.

Repeated measures ANOVAs with a Greenhouse–Geisser correction were conducted to assess whether receiving the training would improve care staff’s person-centredness and hope at T2 and T3 compared to T1. As above, time was the within-subjects condition, and care home was the between-subjects condition. No significant main effects were found for person-centredness for either time (*F*(1.72, 106) = .154, *p* = .83, $\eta_{p}^{2}=.003$ ) or care home (*F*(4, 53) = 1.29, *p* = .29, $\eta_{p}^{2}=.09$ ). The interaction between time and care home was also found to be non-significant (*F*(6.88, 106) = 1.06, *p* = .40, $\eta_{p}^{2}=.07$ ), suggesting that receiving the training session did not result in significantly increased person-centredness.

A significant main effect of time was found for hope (*F*(1.75, 106) = 6.46, *p* = .004, $\eta_{p}^{2}=.109$ ). Bonferroni’s post hoc tests found hope scores to have significantly increased at T2 in comparison to T1 (*p* < .001). At T3, hope scores significantly reduced in comparison to T2 (*p* = .03). No significant differences were found between T1 and T3 (*p* = 1.00). No significant main effect was found for care home (*F*(4, 53) = 1.11, *p* = .36, $\eta_{p}^{2}=.08$ ), with the interaction between time and care home also non-significant (*F*(6.98, 106) = .80, *p* = .06, $\eta_{p}^{2}=.06$ ), suggesting that receiving the training resulted in immediate improvement in hope scores, but this effect was decreased over time.

A series of paired-samples *t*-tests was conducted to assess the effect of the training intervention on reducing burnout at 3 months follow-up compared to baseline. No significant differences were found on scores between any of the three dimensions of burnout: emotional exhaustion (*M*_baseline_ = 18.54, (*SD*) = 10.96; *M*_3months_ = 17.28, *SD* = 11.53; *t*(55) = .90, *p* = .38), depersonalisation (*M*_baseline_ = 4.24, *SD* = 4.50; *M*_3months_ = 4.04, *SD* = 4.02; *t*(53) = .33, *p* = .75) and personal accomplishment (*M*_baseline_ = 34.60, *SD* = 9.74; *M*_3months_ = 36.25, *SD* = 8.34; *t*(47) = −1.34, *p* = .19) 3 months after the training when compared to baseline.

## Discussion and implications

The MEDREV study aimed to deliver and assess the feasibility of a psychological intervention developed to prepare care staff for the management of behaviours that challenge among people with dementia following a review of medications. The ‘Inside Out’ intervention training workshop was executed smoothly and received positive feedback. Post-intervention comments indicated that care staff enjoyed participating in the workshop and both staff and managers were highly satisfied with its content, structure and facilitation by someone with professional experience of the residential care sector.

Confirming other research, MEDREV found that carers require training in the use of medication and the management of behaviours that challenge (Grace and Horstmanshof, 2019; Maidment et al., 2017). Person-centred care is frequently advocated as an approach to improve the communication between care home residents with dementia and formal carers (Morris et al., 2018). Like other research, the VIPS model applied in MEDREV helped care staff implement person-centred care (Røsvik et al., 2011). Other research has found that lack of staff time and resources can be barriers to the appropriate management of behaviour that challenges and care home staff may prioritise tasks, such as administering medication, over providing person-centred care (Nunez et al., 2018; Smythe et al., 2016). Lack of appropriate training may also be a barrier to person-centred care, as we found in MEDREV (Houghton et al., 2016; Morris et al., 2018; Nunez et al., 2018; Smythe et al., 2016).

The intervention did not have a significant effect on overall ADQ attitudes to dementia and person-centredness. However, hope scores did significantly increase immediately post-intervention, when compared to baseline. Although hope scores recorded 3 months post-intervention had reduced to levels similar to those recorded at baseline, this is a promising finding and supports care staff’s accounts that the workshop had *‘refreshed’* their understanding of the capabilities of people with dementia. These findings were underpowered and should be interpreted with caution; further work is required to determine whether these exploratory findings represent this sample. Although attempts were made to recommend reference to the VIPS at handover, in staff meetings and in supervision, limited resources meant that we were unable to systematically monitor whether recommendations were put into practice. Such actions would help ‘keep the message alive’ and are required for longer lasting impact (Michie et al., 2015).

Despite ADQ scores implying no long-standing impact on attitudes towards dementia, positive changes to practice were reported, albeit in a very small sample. Care staff reporting change were more likely to consider behaviours that challenge to be an expression of unmet need and recognised the positive outcomes that could be achieved by providing person-centred care to care home residents with dementia (Macaulay, 2018). Consequently, there were improved efforts to socially engage with residents post-intervention, which was accompanied by reduced eagerness to seek medication to address behaviours that challenge.

The Maslach Burnout Inventory did not change over time, indicating that the ‘Inside Out’ intervention had no impact on burnout. The MARQUE study also found that training care staff had no impact on burnout (Livingston et al., 2019). The qualitative findings were mixed; providing the rationale for reducing psychotropic medication relieved anxieties, but person-centred care was reported as being emotionally demanding and potentially frustrating without additional support for care staff.

Further intervention to build social opportunities within individual care home settings through additional follow-up visits by the researcher, environmental interventions (e.g. VIPS posters) and systemic interventions (e.g. VIPS integrated into supervisions) may have maintained the impetus created by the training and resulted in longer lasting behaviour change (Hawe, 2015; Martin et al., 2012). Other similar studies have also recommended a more intensive longer duration intervention (Ballard et al., 2008; Livingston et al., 2019). This would require greater input from the research team but more detailed data from observational work would generate much more reliable evidence of change over time.

The experience of using the measures with care staff was challenging. Issues arose around the terminology used in the questionnaires; the use of the term ‘callous’ in the Maslach Burnout Inventory proved particularly challenging as many participants did not understand its meaning. Staff also felt uneasy answering some questions as they felt that it was emotionally or morally challenging to do so. Despite the researcher explaining the importance of answering every question, some left questions blank, meaning there was a large amount of missing data. Although the researcher was present to assist at T1 and T2, T3 was completed in the absence of the researcher, which again, resulted in missing data. Future work would require assistance for each completion of the measure and clear guidance to explain their utility for research findings.

The issues discussed constitute limitations for MEDREV and challenges for future research. Face-to-face meetings in small groups with care staff at the training sessions proved successful as a means for explaining the objectives of the research, which had not been understood from information provided by managers or site initiation visits conducted by the research team. The significance of establishing personable relationships with care home staff was crucial and was facilitated by involvement of a researcher with experience in the sector; the experiential knowledge was highly valued by care staff.

## Conclusion

Overall, this evaluation of a psychological training intervention for professional care staff found some success. According to the qualitative data, the intervention resulted in changed practice and perceptions of people with dementia, their capabilities and the antecedents of behaviours that challenge, and appeared to reduce willingness to resort to medication.

Assessing the effect of the intervention on care staff burnout and attitudes to dementia using standard questionnaires proved challenging. Future research is likely to need more presence from the research team through observational work and a more intensive longer duration intervention to strengthen message delivery.

## Supplemental Material

HP0-19-0091-R2_supplementary_file_23-03-20 – Supplemental material for Preparing care home staff to manage challenging behaviours among residents living with dementia: A mixed-methods evaluation Click here for additional data file. Supplemental material, HP0-19-0091-R2_supplementary_file_23-03-20 for Preparing care home staff to manage challenging behaviours among residents living with dementia: A mixed-methods evaluation by Niyah Campbell, Ian D Maidment, Emma Randle and Rachel L Shaw in Health Psychology Open
