# Supplementary material for: Preparing care home staff to manage challenging behaviours among residents living with dementia: A mixed-methods evaluation
Source: Health Psychol Open. 2020 Jul 3;7(2):2055102920933065. doi: 10.1177/2055102920933065 (PMC8851137; doi:10.1177/2055102920933065)
Supplement: HP0-19-0091-R2_supplementary_file_23-03-20 – Supplemental material for Preparing care home staff to manage challenging behaviours among residents living with dementia: A mixed-methods evaluation [file HP0-19-0091-R2_supplementary_file_23-03-20.pdf]

Supplementary file 1: Outline semi-structured schedules for pre- and post-intervention interviews

Interview schedule for care home staff

Pre-intervention

1. Tell me about a typical day in your care home?
2. How often do you deal with what you might call challenging behaviour? What types of behaviour have you experienced?
3. How do you manage challenging behaviour usually? Is there one thing you find that works? Are there different ways of dealing with different types of behaviour?
4. Have you heard about national initiatives to reduce the prescribing of anti-psychotics (sedatives)? What is your opinion of these initiatives?
5. Without telling me who the individual is, can you describe a time when you've looked after someone that you found particularly difficult to care for?
6. Without telling me who the individual is, can you describe a time when you tried something out and it worked really well? Have you now tried this with others/in other situations?
7. What training have you received to help you care for people with dementia?
8. Tell me what you know about dementia from your experience of working here (or elsewhere)
9. What support do you get at work when you experience difficult behaviour?
10. What (if anything) would you like to get out of the training you will receive from us?
11. As part of our work, residents with dementia will have their medication reviewed by a pharmacist. What do you think about this?
12. Is there anything else you would like to say?

Post-intervention

1. What did you think about the training you received?
2. Can you tell me one thing you learned about caring for people with dementia from the training?
3. Can you remember any of the techniques covered in the training? If yes: Have you tried to use these with residents since? How did it go? How easy was it to put into practice? If no: How do you manage people with challenging behaviour generally?
4. Can you tell me one thing you learned about yourself as a carer of people with dementia following the training?
5. Do you feel confident working with people with dementia?

6. How would you describe challenging behaviour? What do you do when faced with challenging behaviour? Is this different from what you did before? How?
7. Have you noticed any difference in the behaviour of the residents you care for?
8. Is there anything else (further training/information) you would like to help with your day-to-day caring role?
9. What did you think about the care home taking part in this project?
10. What did you think about the review of resident's medication carried out by the pharmacist? Do you think it was helpful? (If yes – why, if no, why not?)
11. (If applicable) What did you think about the rating scales e.g. how easy were the scales to use?
12. In some research studies, carers in some care homes would receive training whilst carers in other homes would receive no training to test how useful the training is. How would you feel about taking part in a study like this where you were one of the carers who didn't receive any training?
13. Is there anything else you'd like to say?

<u>Interview schedule for care home managers</u>

<u>Pre-intervention</u>

1. Can you tell me about your professional background? How long have you been manager here? Where were you previously?
2. Have you received specialist training for caring for people with dementia?
3. What is your policy for managing challenging behaviours?
4. What is your policy regarding medicating for challenging behaviours?
5. Have you heard about national initiatives to reduce the prescribing of anti-psychotics (sedatives)? What is your opinion of these initiatives?
6. What training do you provide to care home staff caring for people with dementia?
7. What support do you offer on a general basis for your staff?
8. What do you anticipate the barriers to implementing this intervention will be in your care home?
13. What, if anything, are you hoping to get out of the training?
14. As part of our work, residents with dementia will have their medication reviewed by a pharmacist. What do you think about this?
15. Is there anything you would like to say?

<u>Post-intervention</u>

1. What has it been like running this training at your care home?
2. What do you think the staff thought about it? What did you think about it?

3. Have you noticed any changes in how staff behave with the people with dementia they care for following the training?
4. Has there been any noticeable difference in residents' challenging behaviour since the intervention?
5. What has been the impact of the reduction in medication?
6. Have the techniques to help manage challenging behaviour helped?
7. Do you think it will be useful to include this training in your staff induction?
8. What did you think of the medication review carried out by the pharmacist? Do you think it was beneficial?
9. (If applicable) How easy was it to implement any changes to medication suggested by the pharmacist?
10. How easy was it to implement the training for carers in your care home?
11. What did you think about the rating scales e.g. how easy for the staff were the scales to use?
12. Some research studies involve some care homes receiving training and the medication review, whilst other homes don't receive any to test how useful the training and the review is. How would you feel about taking part in a study like this where your home was in the group who didn't receive any training?
13. Is there anything else you'd like to say?

<u>Interview schedule for GPs</u>

<u>Pre-intervention</u>

1. Tell me about your approach to people with dementia generally in your practice.
2. What do you know about the NICE / National Dementia Strategy guidance regarding antipsychotics for people with dementia?
3. What is your opinion on this guidance? Has it affected your prescribing? Are you using now different medicines rather than anti-psychotics? If, yes, which medications?
4. What else do you know about the guidance for best practice to manage challenging behaviour in people with dementia?
5. How is your relationship with the community pharmacists involved in the practice with X care home?
6. Can you tell me about your dealings with X care home? How often are you called out/deal with families of residents? Do you visit the care home?
7. Do you need any kind of support in your dealings with X care homes?
8. What, if anything, are you hoping to get out of the training?
9. Is there anything else you'd like to say?

Post-intervention

1. What did you think of the training you received?
2. Have you noticed any difference in the cases being brought to you from X care home (related to dementia and challenging behaviour)?
3. Can you tell me about any interactions with care home staff or families about reducing medication and challenging behaviour since you received the training? How did it go? Did the training help?
4. Do you know more about the NICE / National Dementia Strategy guidance to reduce antipsychotics? Have you been able to use this knowledge to help explain things to patients?
5. What do you know about the specialist guidance on how to manage challenging behaviour? Have you been able to use this knowledge in consultation with families/care home staff?
6. What do you think about the best practice guidance?
7. Do you need any support in dealing with the families and care home staff in relation to people with dementia and challenging behaviour?
8. What did you think of the medication review carried out by the pharmacist? Do you think it was beneficial?
9. How easy was it to implement any changes to patient's medication suggested by the pharmacist after the medication review? What if anything would have made it easier to implement the changes?
10. In the future, this work may progress to a randomised controlled trial. How would you feel about being allocated to a control group in a randomised controlled trial to assess how effective the intervention (training programme and medication review) was?
11. Is there anything else you'd like to say?
